# Knowledge, attitude, and practice towards bacterial multidrug-resistance and structural equation modeling analysis among intensive care unit nurses and physicians

**DOI:** 10.1371/journal.pone.0304734

**Published:** 2024-06-14

**Authors:** Zhongping Ai, Yaping Fang, Xiaolan Gao, Li Wang, Min Yu

**Affiliations:** 1 Nursing Department, The Affiliated Hospital of Southwest Medical University, Luzhou, Sichuan, China; 2 ICU, The Affiliated Hospital of Southwest Medical University, Luzhou, Sichuan, China; Zarqa University, JORDAN

## Abstract

**Background:**

The intensive care unit (ICU) is a department with a high risk of MDR bacteria, and ICU nurses and physicians play critical roles in bacterial multidrug resistance (MDR) prevention.

**Objectives:**

To explore the knowledge, attitudes, and practice (KAP) towards bacterial MDR among ICU nurses and physicians.

**Methods:**

A self-designed questionnaire was administered to collect data. Structural equation modeling (SEM) was applied to assess the associations among study variables.

**Results:**

A total of 369 questionnaires were collected; 43 questionnaires were excluded due to self-contradictory on the trap question or the obviously repeated pattern. Finally, 326 (88.35%) valid questionnaires were included in the analysis. The knowledge, attitudes, and practice were 13.57 ± 1.69 (90.47%, possible range: 0–15), 38.75 ± 2.23 (96.88%, possible range: 8–40), and 47.40 ± 3.59 (94.80%, possible range: 10–50). The SEM showed that knowledge had a direct effect on attitude with a direct effect value of 0.61 (P < 0.001) and a direct negative effect on practice with a direct effect value of -0.30 (P = 0.009). The direct effect of attitude on practice was 0.89 (P < 0.001); the indirect effect of knowledge through attitude on practice was 0.52 (P < 0.001). Job satisfaction had a direct effect on attitude and practice, with an effect value of 0.52 (P = 0.030) and 0.75 (P = 0.040). Being a physician (OR = 0.354, 95%CI: 0.159–0.790, P = 0.011), 5–9.9 years of practice (OR = 4.534, 95%CI: 1.878–8.721, P < 0.001), and ≥ 10 years of practice (OR = 3.369, 95%CI: 1.301–8.721, P = 0.012) were independently associated with good knowledge. The attitude scores (OR = 1.499, 95%CI: 1.227–1.830, P < 0.001), male gender (OR = 0.390, 95%CI: 0.175–0.870, P = 0.022), and 5–9.9 years of experience (OR = 0.373, 95%CI: 0.177–0.787, P = 0.010) were independently associated with proactive practice.

**Conclusion:**

Nurses and physicians in the ICU showed good knowledge, positive attitudes, and proactive practice toward bacterial MDR. Nurses and physicians’ knowledge had a direct effect on their attitude, while attitude might directly influence the practice and also play a mediating role between knowledge and practice. Job satisfaction might directly support the positive attitude and practice toward bacterial MDR.

## Introduction

Multiple-drug resistance (MDR) is antimicrobial resistance shown by a species of microorganism to at least one antimicrobial drug in three or more antimicrobial categories [1]. Bacterial MDR has profound economic and public health impacts [2, 3]. Prevention and management of MDR is a complex process with a significant risk of failure, which might lead to poor prognosis when it occurs [4]. The intensive care unit (ICU) is a department with a high risk of MDR bacteria. Indeed, the main risk factors for being infected with an MDR bacteria include known colonization with resistant bacteria, previous infections, previous exposure to broad-spectrum antibiotics, advanced comorbidities, prolonged hospital stay, mechanical ventilation, and catheter placement (urinary catheters and central venous catheters), all of which are commonly encountered in ICU patients [5, 6]. ICU nurses and physicians are at the frontline of clinical care provided to ICU patients and play critical roles in preventing bacterial MDR by applying the strict principles of MDR prevention, including antimicrobial stewardship, hand hygiene, microbial screening, isolation precautions, empiric isolation, decolonization, skin antisepsis, transmission prevention from the ICU environment, and transmission prevention of known MDR bacteria (e.g., methicillin-resistant *Staphylococcus aureus*, vancomycin-resistant *Enterococcus faecium*, MDR Gram-negative bacteria, etc.) [7, 8]. Thus, the knowledge, attitude, and practice of ICU medical staff towards bacterial MDR might be paramount to reducing the incidence.

Knowledge, attitudes, and practice (KAP) survey is a structured method to identify the gaps and misconceptions that are barriers to the correct and optimal implementation of a given subject in a given population [9]. The results of KAP studies can be used to design education, training, and continuous education interventions to improve the KAP. Several studies around the globe reported considerably insufficient KAP regarding MDR in healthcare providers [10–12], including in the ICU [13–15]. Indeed, the KAP towards MDR has been shown to be poor among medical students in East China [10]. The KAP towards proper antibiotic use and MDR was poor in Tanzania [11]. Similar results were reported in Pakistan [12]. Regarding the ICU, where MDR prevention is paramount, a study showed that there was room for improvement in neonatal ICUs in Zhejiang, China [15]. Similar results were reported in ICUs in New York (United States of America) [13]. On the other hand, most ICU physicians in Europe consider MDR to be a substantial problem [14]. Nevertheless, studies on the KAP of ICU nurses and physicians in China are limited.

Identifying the KAP gaps and improving the KAP in ICU nurses and physicians is essential since they are at the forefront of MDR prevention and management. Therefore, this study explored the KAP towards bacterial MDR among ICU nurses and physicians.

## Materials and methods

### Study design and participants

This multicenter cross-sectional study enrolled ICU nurses and physicians from the twelve hospitals in China between Aug 2023 and Dec 2023. Physicians and nurses with professional qualifications were included. The nurses and physicians rotating among different departments and the interns were excluded. The study was approved by the Ethics Committee of the Affiliated Hospital of Southwest Medical University (KY2023199). Written informed consent was obtained from the participants before the study.

### Questionnaire and quality control

The questionnaire was designed with reference to the relevant published literature [15–17], “China experts’ consensus on prevention and control of multidrug-resistant organism healthcare-associated infection” [18], and “Recommendations and guidelines for the treatment of infections due to multidrug-resistant organisms” [19]. A pilot test (n = 29) was conducted, with a Cronbach’s α of 0.74, indicating good reliability of the questionnaire.

The final questionnaire was in Chinese and included 43 items. The demographic information consisted of 9 items, of which the job satisfaction was assessed by participants on a five-point Likert scale ranging from “very dissatisfied” to “very satisfied”. The knowledge section consisted of 16 items, the attitude consisted of 8 items, and the practice consisted of 10 items. For the knowledge items, 1 point was scored for a correct answer and 0 points for a wrong or unclear answer. Item K11 was a non-assigned trap question used only to eliminate the illogical questionnaire. The possible total score for knowledge ranged from 0 to 15. The attitude and practice items were scored on a five-point Likert scale. Specifically, the attitude items were scored from “strongly agree” (5) to “strongly disagree” (1), except that item A1 was assigned a reverse score, with the possible score range of 8–40. The practice items were all scored from “totally compliantly” (5) to “not compliantly at all” (1), with the possible score range of 10–50. The knowledge, attitude, and practice scores equal to or greater than 80% (12, 32, and 40, respectively) were considered as good knowledge, positive attitude, and proactive practice, respectively; 60–79% as moderate and less than 60% as poor knowledge, negative attitude, and inappropriate practice, respectively [20].

The data were collected using the electronic questionnaire on the *Sojump* online platform in China, which was distributed to the WeChat group of ICU nurses and physicians. If a participant encountered a problem when completing the questionnaire, the research team members were responsible for answering the problems in a timely manner. When data collection was completed, the quality of the questionnaires would be checked. Questionnaires with an answer time of < 120 s, obvious logical errors, or choosing the same option thoroughly were considered invalid.

### Statistical analysis

Stata 17.0 (Stata Corporation, College Station, TX, USA) was used for statistical analysis. The continuous variables were expressed using mean ± standard deviation (SD) and analyzed using Student’s t-test (two groups) or one-way ANOVA (more than two groups). The categorical variables were expressed as numbers and percentages [n (%)]. Pearson’s correlation analysis was used to analyze the correlations among knowledge, attitude, and practice. Univariable and multivariable logistic regression analyses were used to identify factors associated with good knowledge, positive attitude, and proactive practice. These were defined as scores exceeding the median [21]. Structural equation modeling (SEM) was used to test the following hypotheses: (H1) knowledge would have a direct effect on practice; (H2) knowledge would have an indirect effect on practice through attitude; (H3) knowledge would have an indirect effect on practice through job satisfaction; (H4) professional title would have an effect on knowledge, attitudes, and practice; and (H5) ICU work experience would have an effect on attitude, practice, professional title, and job satisfaction. Two-sided P < 0.05 were considered statistically significant.

## Results

A total of 369 questionnaires were collected; 43 questionnaires were excluded due to self-contradictory on the trap question or the obviously repeated pattern. Thus, 326 (88.35%) valid questionnaires were included in the analysis. Most participants were female (86.81%), nurses (89.26%), 30–35 years old (41.10%), college/undergraduate (91.72%), with a junior or below professional title (60.12%), with ≥ 10 years of work experience (38.65%), and felt satisfied with current work (66.56%) **(Table 1)**.

**Table 1:** Characteristics of the participants.

Characteristics	n (%)	Knowledge		Attitude		Practice	
		Mean ± SD	P	Mean ± SD	P	Mean ± SD	P
**Total**	326	13.57 ± 1.69		38.75 ± 2.23		47.40 ± 3.59	
**Gender**			0.352		0.163		0.005
Male	43 (13.19)	13.79 ± 1.17		38.30 ± 2.10		45.98 ± 3.58	
Female	283 (86.81)	13.53 ± 1.75		38.81 ± 2.25		47.61 ± 3.55	
**Age (years old)**			0.004		0.677		0.307
< 30	125 (38.24)	13.18 ± 1.69		38.72 ± 1.95		47.64 ± 3.41	
30–35	134 (41.10)	13.78 ± 1.78		38.66 ± 2.80		47.03 ± 3.99	
> 35	67 (20.55)	13.88 ± 1.33		38.96 ± 1.24		47.67 ± 3.01	
**Education**			< 0.001		0.135		0.168
High school/technical secondary school	3 (0.92)	8.67 ± 4.73		36.67 ± 5.77		46.00 ± 6.93	
College/undergraduate	299 (91.72)	13.55 ± 1.62		38.81 ± 2.12		47.51 ± 3.34	
Master/PhD	24 (7.36)	14.46 ± 0.78		38.25 ± 2.94		46.17 ± 5.62	
**Hospital**			< 0.001		0.035		0.242
Public primary/secondary	64 (19.63)	12.88 ± 2.46		38.13 ± 3.62		47.80 ± 3.51	
Public tertiary	249 (76.38)	13.80 ± 1.28		38.92 ± 1.65		47.23 ± 3.61	
Private hospital	13 (3.99)	12.46 ± 2.57		38.46 ± 2.73		48.62 ± 3.38	
**Occupation**			0.108		0.383		0.722
Nurse	291 (89.26)	13.52 ± 1.73		38.71 ± 2.33		47.37 ± 3.64	
Physician	35 (10.74)	14.00 ± 1.16		39.06 ± 1.19		47.60 ± 3.18	
**Professional title**			< 0.001		0.829		0.750
Junior and below	196 (60.12)	13.27 ± 1.88		38.68 ± 2.46		47.51 ± 3.56	
Intermediate	108 (33.13)	14.04 ± 1.24		38.84 ± 1.94		47.24 ± 3.75	
Vice senior/senior	22 (6.75)	13.91 ± 1.06		38.82 ± 1.18		47.09 ± 3.04	
**Years of clinical practice**			0.005		0.449		0.917
< 3	49 (15.03)	13.02 ± 2.01		38.35 ± 2.28		47.20 ± 3.79	
3–4.9	49 (15.03)	13.44 ± 1.37		38.92 ± 1.67		47.61 ± 3.53	
5–9.9	102 (31.29)	13.41 ± 2.06		38.92 ± 1.67		47.27 ± 3.62	
≥ 10	126 (38.65)	13.95 ± 1.19		38.90 ± 1.68		47.48 ± 3.55	
**Years of ICU practice**			< 0.001		0.149		0.068
< 1	77 (23.62)	12.68 ± 2.33		38.23 ± 3.32		47.79 ± 3.73	
1–2.9	44 (13.50)	13.34 ± 1.94		38.55 ± 2.32		47.27 ± 3.90	
3–4.9	58 (17.79)	13,64 ± 1.04		38.91 ± 1.41		47.53 ± 3.01	
5–9.9	68 (20.86)	14.12 ± 1.11		38.99 ± 2.11		46.32 ± 4.34	
≥ 10	79 (24.23)	14.04 ± 1.16		39.03 ± 1.18		47.90 ± 2.72	
**Job satisfaction**			0.039		0.277		0.002
Very satisfied	101 (30.98)	13.19 ± 1.67		38.77 ± 2.03		48.51 ± 2.61	
Relatively satisfied	116 (35.58)	13.73 ± 1.39		38.91 ± 1.34		47.06 ± 3.10	
Moderate satisfied	94 (28.83)	13.68 ± 2.03		38.67 ± 2.86		46.73 ± 4.13	
Dissatisfactory	15 (4.60)	14.13 ± 0.99		37.73 ± 3.99		46.60 ± 6.66	

The knowledge score was 13.57 ± 1.69 (90.47%, possible range: 0–15), indicating good knowledge. Knowledge scores varied in age groups (P = 0.004), education (P < 0.001), professional titles (P < 0.001), work experience (P = 0.005), and job satisfaction (P = 0.039) **(Table 1)**. The correct rate of all knowledge items was higher than 80%, except for K1 (51.23%), the item about the definition of MDR bacteria **(S1 Table in S1 File)**. The attitude score was 38.75 ± 2.23 (96.88%, possible range: 8–40), indicating positive attitudes. Higher attitude scores were observed in public tertiary hospitals (P = 0.035). More than 98% of nurses and physicians agreed that “Maintaining proper hand hygiene among medical staff is crucial for the prevention and control of multidrug-resistant bacteria infections” (A2) and that “Each medical staff should actively participate in the prevention and control of multidrug-resistant bacteria infections” (A5) **(S2 Table in S1 File)**. The practice score was 47.40 ± 3.59 (possible range: 94.80%, 10–50), indicating proactive practices. Higher practice scores were observed in females (P = 0.005) and in people satisfied with their current work (P = 0.002). More than 98% of participants reported that they would wear gloves when coming into contact with wounds, blood, body fluids, drainage fluid, and secretions from patients with multidrug-resistant infections and would wash or disinfect their hands immediately after removing gloves (P4) **(S3 Table in S1 File)**.

The knowledge scores were positively correlated with the attitude scores (r = 0.442, P < 0.001), and the attitude scores were positively correlated with the practice scores (r = 0.501, P < 0.001). The knowledge and practice scores were not correlated (r = 0.094, P = 0.090).

For the purpose of the univariable and multivariable analyses, the median values of knowledge, attitude, and practice were 14 (106 participants >14, 220 participants ≤14), 39 (142 participants >39, 184 participants ≤39), and 49 (139 participants >49, 187 participants ≤49), respectively, in the present study.

Being a physician (OR = 0.354, 95%CI: 0.159–0.790, P = 0.011), 5–9.9 years of practice (OR = 4.534, 95%CI: 1.878–8.721, P < 0.001), and ≥10 years of practice (OR = 3.369, 95%CI: 1.301–8.721, P = 0.012) were independently associated with good knowledge (**Table 2**). Male gender (OR = 0.347, 95%CI: 0.165–0.730, P = 0.005) was the only factor associated with positive attitude (**Table 3**). The attitude scores (OR = 1.499, 95%CI: 1.227–1.830, P < 0.001), male gender (OR = 0.390, 95%CI: 0.175–0.870, P = 0.022), and 5–9.9 years of experience (OR = 0.373, 95%CI: 0.177–0.787, P = 0.010) were independently associated with proactive practice (**Table 4**).

**Table 2:** Analysis of the factors associated with good knowledge.

Knowledge	Univariable logistic regression		Multivariable logistic regression	
	OR (95%CI)	P	OR (95%CI)	P
**Gender**				
Male	1.002 (0.505–1.988)	0.995		
Female	ref			
**Age (years old)**				
< 30	0.459 (0.244–0.865)	**0.016**	0.975 (0.350–2.713)	0.961
30–35	0.752 (0.412–1.373)	0.354	0.784 (0.350–1.759)	0.556
> 35	ref		ref	
**Hospital**				
Public primary/secondary	1.540 (0.305–7.774)	0.601		
Public tertiary	3.113 (0.675–14.358)	0.145		
Private hospital	ref			
**Occupation**				
Nurse	ref		ref	
Physician	0.467 (0.230–0.947)	**0.035**	0.354 (0.159–0.790)	**0.011**
**Professional title**				
Junior and below	ref		ref	
Intermediate	2.134 (1.300–3.501)	**0.003**	1.400 (0.733–2.675)	0.309
Vice senior/senior	1.292 (0.499–3.347)	0.597	0.596 (0.169–2.101)	0.421
**Years of clinical practice**				
< 3	ref			
3–4.9	1.000 (0.408–2.452)	1.000		
5–9.9	1.209 (0.564–2.590)	0.625		
≥ 10	1.762 (0.851–3.651)	0.127		
**Years of ICU practice**				
< 1	ref		ref	
1–2.9	2.297 (0.962–5.487)	0.061	2.423 (0.980–5.991)	0.055
3–4.9	1.422 (0.603–3.355)	0.421	1.397 (0.564–3.461)	0.471
5–9.9	4.376 (2.040–9.386)	**<0.001**	4.534 (1.878–10.947)	**<0.001**
≥ 10	3.720 (1.767–7.829)	**<0.001**	3.369 (1.301–8.721)	**0.012**
**Job satisfaction**				
Very satisfied	0.300 (0.098–0.922)	**0.036**	0.374 (0.114–1.225)	0.104
Relatively satisfied	0.724 (0.246–2.135)	0.559	0.723 (0.230–2.267)	0.578
Moderate satisfied	0.618 (0.206–1.856)	0.391	0.553 (0.174–1.761)	0.316
Dissatisfactory	ref		ref	

**Table 3:** Analysis of the factors associated with positive attitude.

Attitude	Univariable logistic regression	
	OR (95%CI)	P
**Knowledge**	1.135 (0.982–1.312)	0.085
**Gender**		
Male	0.347 (0.165–0.730)	**0.005**
Female	ref	
**Age (years old)**		
< 30	1.025 (0.562–1.871)	0.935
30–35	1.164 (0.643–2.105)	0.616
> 35	ref	
**Hospital**		
Public primary/secondary	ref	
Public tertiary	1.157 (0.662–2.021)	0.609
Private hospital	1.253 (0.378–4.156)	0.713
**Occupation**		
Nurse	ref	
Physician	0.907 (0.448–1.834)	0.785
**Professional title**		
Junior and below	ref	
Intermediate	1.062 (0.662–1.704)	0.802
Vice senior/senior	0.597 (0.233–1.529)	0.282
**Years of clinical practice**		
< 3	ref	
3–4.9	1.653 (0.738–3.705)	0.222
5–9.9	1.472 (0.731–2.961)	0.279
≥ 10	0.520 (1.250–2.468)	0.520
**Years of ICU practice**		
< 1	ref	
1–2.9	1.258 (0.592–2.673)	0.551
3–4.9	1.168 (0.582–2.345)	0.662
5–9.9	1.755 (0.905–3.404)	0.096
≥ 10	1.317 (0.694–2.497)	0.400
**Job satisfaction**		
Very satisfied	1.120 (0.378–3.322)	0.837
Relatively satisfied	0.724 (0.246–2.135)	0.559
Moderate satisfied	0.847 (0.284–2.527)	0.765
Dissatisfactory	ref	

**Table 4:** Analysis of the factors associated with good practice.

Practice	Univariable logistic regression		Multivariable logistic regression	
	OR (95%CI)	P	OR (95%CI)	P
**Knowledge**	1.014 (0.889–1.156)	0.836		
**Attitude**	1.444 (1.190–1.754)	**<0.001**	1.499 (1.227–1.830)	**<0.001**
**Gender**				
Male	0.362 (0.172–0.762)	**0.007**	0.390 (0.175–0.870)	**0.022**
Female	ref		ref	
**Age (years old)**				
< 30	0.864 (0.477–1.565)	0.629		
30–35	0.594 (0.328–1.076)	0.086		
> 35	ref			
**Hospital**				
Public primary/secondary	ref		ref	
Public tertiary	0.810 (0.466–1.409)	0.456	0.696 (0.372–1.301)	0.256
Private hospital	4.023 (1.011–16.005)	**0.048**	4.042 (0.828–19.747)	0.084
**Occupation**				
Nurse	ref			
Physician	0.764 (0.379–1.543)	0.453		
**Professional title**				
Junior and below	ref			
Intermediate	1.027 (0.639–1.650)	0.911		
Vice senior/senior	0.762 (0.306–1.900)	0.560		
**Years of clinical practice**				
< 3	ref			
3–4.9	1.283 (0.577–2.853)	0.542		
5–9.9	0.975 (0.487–1.950)	0.942		
≥ 10	1.123 (0.575–2.194)	0.734		
**Years of ICU practice**				
< 1	ref		ref	
1–2.9	0.667 (0.317–1.407)	0.288	0.705 (0.315–1.579)	0.395
3–4.9	0.462 (0.229–0.933)	**0.031**	0.477 (0.221–1.029)	0.059
5–9.9	0.392 (0.198–0.776)	**0.007**	0.373 (0.177–0.787)	**0.010**
≥ 10	0.814 (0.434–1.526)	0.521	0.927 (0.455–1.891)	0.835
**Job satisfaction**				
Very satisfied	1.280 (0.431–3.806)	0.656		
Relatively satisfied	0.426 (0.144–1.263)	0.124		
Moderate satisfied	0.473 (0.158–1.421)	0.182		
Dissatisfactory	ref			

**Fig 1:**
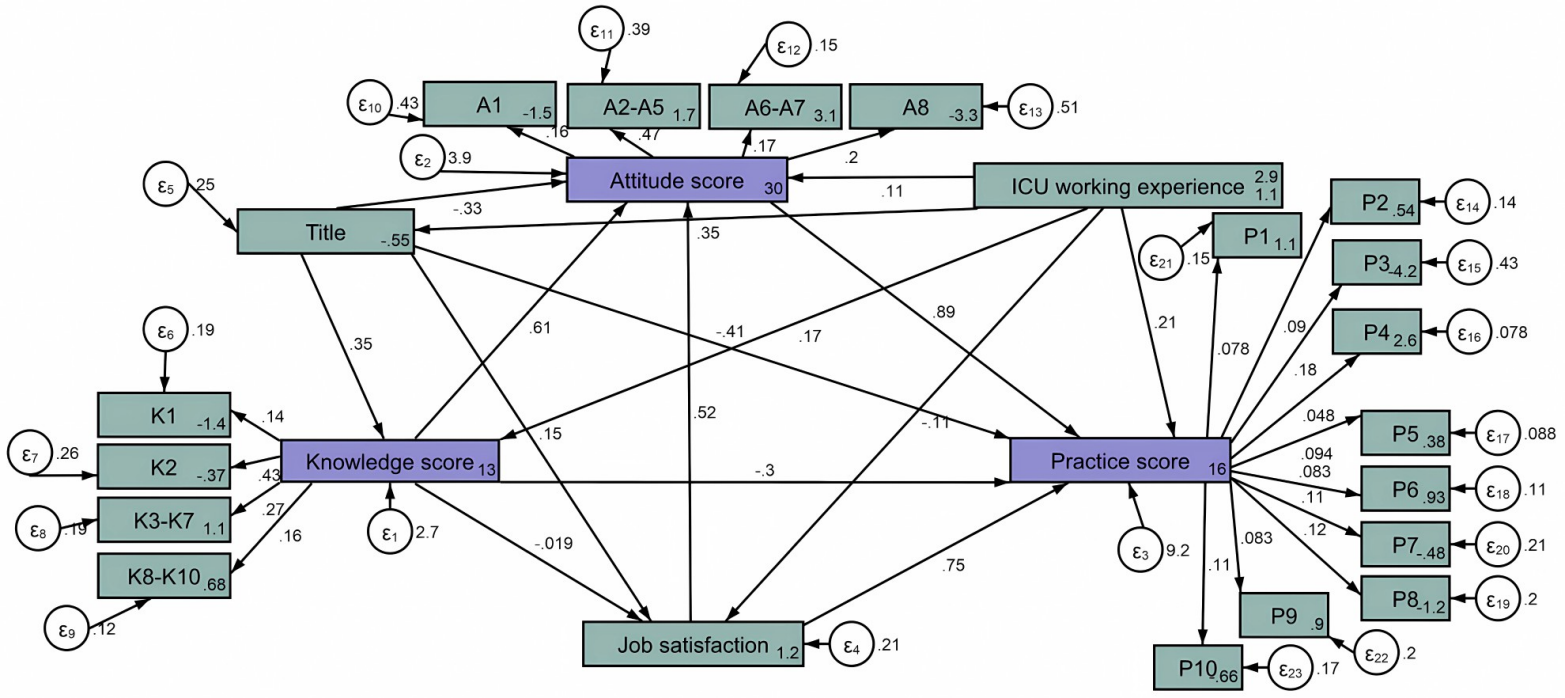
Structural equation modeling. Green represents the observed variables, and purple represents the latent variables.

The SEM showed that knowledge had a direct effect on attitude with a direct effect value of 0.61 (P < 0.001) and a direct negative effect on practice with a direct effect value of -0.30 (P = 0.009). The direct effect of attitude on practice was 0.89 (P < 0.001); the indirect effect of knowledge through attitude on practice was 0.52 (P < 0.001), indicating that attitude plays a mediating role between knowledge and practice. Job satisfaction had a direct effect on attitude and practice, with an effect value of 0.52 (P = 0.030) and 0.75 (P = 0.040) **(Fig 1 and S4 Table in S1 File)**.

## Discussion

This study suggested that nurses and physicians in ICUs have good knowledge, positive attitudes, and active practice toward bacterial MDR. Nurses and physicians’ knowledge had direct effects on their attitude and practice, and attitude also played a mediating role between knowledge and practice. The results might provide a theoretical basis for the continuing education of nurses and doctors.

ICU nurses and physicians are the primary actors who can implement preventive and management measures against MDR infections. Thus, their KAP towards MDR bacteria and infections is paramount to the proper control of MDR bacteria. Previous studies showed that the KAP towards MDR bacteria among healthcare providers was poor or moderate [11, 12, 22–24]. A Swedish study reported that registered nurses had insufficient knowledge, behavior, and emotional response to patients with MDR bacteria, but they considered that it was their responsibility to adhere to preventive measures for infection control [22]. The knowledge of clinicians in Pakistan is relatively poor regarding antibiotic resistance and drugs of choice for certain infections [12]. On the other hand, the KAP towards antibiotic use of physicians in Ghana was widely variable [24]. For ICU nurses and physicians, a study performed on ICU physicians in New York (USA) showed that only 33% were aware of the use of antibiotic susceptibility testing, and only a small number were aware of the resistance mechanisms [13]. Only 24.2% of the ICU respondents in Europe thought that MDR bacteria represented a major problem [14], and only 25% of respondents in America were confident about using antibiotics correctly in the ICU [25]. In this study, the correct rate of all knowledge items was over 80%, except for the definition of MDR bacteria. The results were supported by Min et al. [10], who reported that most statements about MDR were answered correctly by the medical students but that gaps were still observed in the issues of antimicrobial targets and bacterial transmission. Although the knowledge score was high, the correct rates were not 100%, indicating that some education and training remain to be done, and the results of the present study could be used to refine the existing training programs on MDR.

Min et al. [10] reported that overconfident attitudes and inappropriate behaviors regarding antimicrobial overuse and misuse were observed in medical students. For example, some of them declared that there is no risk of MDR as long as the antimicrobials are taken correctly, and several participants reported buying antimicrobials from friends or family members to treat the same illness. This study reported good knowledge, positive attitudes, and proactive practice among ICU physicians and nurses in China. The results were supported by a previous study in neonatal ICUs (NICUs), with high KAP scores towards prevention and control of nosocomial infection with MDR organisms [15]. These results highlight the need for cultivating proper attitudes and practices. In the present study, attitudes were positive, but some aspects still needed improvements, including the risk of carrying MDR bacteria back home and the need for wearing a face shield when there is a risk of being exposed to droplets or aerosols from patients with MDR infections. The ICU is a breeding ground for MDR bacteria [7, 26], and preventing them from exiting the ICU and spreading in the community is important to prevent large-scale MDR in the community. That particular point should be improved in future training, and strict decontamination and cleaning protocols should be emphasized.

The good knowledge, positive attitude, and proactive practice towards MDR among ICU physicians and nurses in China could be explained by the relatively recent implementation of strict nationwide policies regarding MDR management. Indeed, China is facing a major MDR crisis that threatens public health and the economy [27–29]. The Chinese government designed and implemented strict policies regarding antibiotic stewardship, hygiene, and sanitation [30–32]. Southwest China is a region with a relatively low socioeconomic status compared with other regions [33]. It is very important to prevent MDR infection to reduce the related costs. This study suggests that these policies are working since they resulted in high KAP scores. Nevertheless, a KAP study from India indicated that novel, innovative antibiotics are needed for the management of MDR Gram-negative infections [34]. Hence, an adequate mixture of novel antibiotics judiciously used and adequate infection control methods could help manage MDR infections. This should be extended to anyone taking care of patients, as a recent study in Ecuador revealed poor KAP of caretakers regarding the appropriate use of antibiotics [35].

The multivariable analysis showed that being a physician was negatively associated with knowledge. Although physicians are the ones prescribing antibiotics, they minimally interact with patients compared to nurses. A longer work experience was associated with better KAP toward antibiotics MDR. The male gender was also negatively associated with the attitude and practice scores. Although differences in KAP toward antibiotic use and misuse were observed in the general population [36, 37], the available data were not about the KAP of MDR in healthcare providers. Additional studies are necessary to examine that issue.

The SEM analysis in this study showed that knowledge influences attitude and practice and that attitude influences practice. Hence, it highlighted the importance of first cultivating good knowledge through proper training at school and providing frequent continuing education for working healthcare providers. Job satisfaction also influenced KAP, highlighting the importance of maintaining a good work attitude and environment. Indeed, job satisfaction influences care delivery and all aspects of the healthcare environment, including sanitation and MDR prevention [38, 39]. Policymakers and hospital leaders should pay attention to maintaining a positive work environment to favor job satisfaction, which might help medical staff pay attention to MDR and clinical practice.

This study had limitations. Firstly, it was performed at only twelve hospitals, which is small considering the number of hospitals in China, leading to low generalizability. Secondly, most participants showed high KAP scores, with the scores clustered in the high ranges, limiting the possible statistical analyses of the relationships among factors. Additionally, as for all KAP studies, the present study might suffer from the social desirability bias, in which the participants might be tempted to answer what is socially acceptable or desirable instead of what they are doing [40, 41]. Further study is needed to verify the findings, and future studies could focus on comparing the changes in KAP with time and policies.

## Conclusions

In conclusion, nurses and physicians in the ICU showed good knowledge, positive attitudes, and proactive practice toward bacterial MDR. Nurses and physicians’ knowledge had a direct effect on their attitude, while attitude might directly influence the practice and also play a mediating role between knowledge and practice. Job satisfaction might directly support the positive attitude and practice toward bacterial MDR.

## Supporting information

S1 File (DOCX)

S1 Questionnaire (DOCX)
