# Supplementary material for: Knowledge, attitude, and practice towards bacterial multidrug-resistance and structural equation modeling analysis among intensive care unit nurses and physicians
Source: PLoS One. 2024 Jun 14;19(6):e0304734. doi: 10.1371/journal.pone.0304734 (PMC11178221; doi:10.1371/journal.pone.0304734)
Supplement: S1 File — (DOCX) [file pone.0304734.s001.docx]

Supplementary **Table S1.** Knowledge

| Items | Correct, n (%) |
| --- | --- |
| K1. The definition of multidrug-resistant bacteria. | 167 (51.23) |
| K2. The main risk factors for multidrug-resistant bacterial infections: | |
| K2.1. Adolescents | 270 (82.82) |
| K2.2. Immunocompromised (including patients with diabetes mellitus, chronic obstructive pulmonary disease, cirrhosis, uremia, and oncology patients on long-term immunosuppressive therapy, radiation, and/or chemotherapy) | 317 (97.24) |
| K2.3. Undergoing invasive procedures, with prolonged tube placement | 306 (93.87) |
| K2.4. Prolonged hospital stays | 309 (94.79) |
| K2.5. With a history of multidrug-resistant bacterial colonization or infection | 298 (91.41) |
| K2.6. Recently (within 90 days) treated with 3 or more antimicrobial drugs | 289 (88.65) |
| K3. Patients and carriers of multidrug-resistant bacterial infections are the main sources of biological transmission of these bacteria in the hospital. | 298 (91.41) |
| K4. Medical devices and the environment that is contaminated with multidrug-resistant bacteria constitute an abiotic source in hospitals. | 302 (92.64) |
| K5. The transmission of multidrug-resistant bacteria in hospitals can be achieved in a variety of ways, while contact transmission being the most common method of transmission in hospitals. | 311 (95.40) |
| K6. Wearing gloves when touching patients with multidrug-resistant infections can prevent hand contamination, and therefore hand washing is not required after removing gloves. | 323 (99.08) |
| K7. Medical staffs should wear a face mask, mask, goggles, and isolation clothing when performing operations that may cause splashing of blood, body fluids, secretions, or excretions. | 319 (97.85) |
| K8. Patients with multidrug-resistant infections or colonization should be placed in a single room as far as possible. If a single room is not available, can patients with the same multi-resistant infection or colonization be placed in the same room? | 275 (84.36) |
| K9. Medical equipment, instruments, and items used by patients with multidrug-resistant infections, should be used exclusively by the patient and disinfected in a timely manner. | 322 (98.77) |
| K10. Medical equipment or instruments used by patients with multi-drug resistant infections, which cannot be dedicated for exclusive use, should be thoroughly wiped and disinfected after each use. | 317 (97.24) |

Supplementary **Table S2. Attitude**

| Items | Strongly agree, n (%) | Relatively agree, n (%) | Neutral, n (%) | Relatively disagree, n (%) | Strongly disagree, n (%) |
| --- | --- | --- | --- | --- | --- |
| A1. Multidrug-resistant infections are common in the ICU and are difficult to avoid, so there is no need to pay particular attention to them. | 7 (2.15) | 3 (0.92) | 6 (1.84) | 45 (13.80) | 265 (81.29) |
| A2. Maintaining proper hand hygiene among medical staff is crucial for the prevention and control of multidrug-resistant bacteria infections. | 316 (96.93) | 6 (1.84) | 1 (0.31) | 1 (0.31) | 2 (0.61) |
| A3. Physicians must adhere to basic principles, implement hierarchical management, and use personalized medication of antibacterial drugs, which is important for prevention and control of multidrug-resistant bacteria infections. | 311 (95.40) | 11 (3.37) | 2 (0.61) | 1 (0.31) | 1 (0.31) |
| A4. Clinical nurses should possess a thorough understanding of multidrug-resistant bacteria and diligently implement measures to isolate, prevent and control their spread, which is important for prevention and control of multidrug-resistant bacteria infections. | 311 (95.40) | 12 (3.68) | 2 (0.61) | 0 | 1 (0.31) |
| A5. Each medical staff should actively participate in the prevention and control of multidrug-resistant bacteria. | 313 (96.01) | 8 (2.45) | 4 (1.23) | 0 | 1 (0.31) |
| A6. It is necessary to strengthen the training and continuing education for clinical nurses regarding multidrug-resistant bacteria. | 309 (94.79) | 12 (3.68) | 4 (1.23) | 0 | 1 (0.31) |
| A7. It is necessary to strengthen the training and continuing education for physicians regarding multidrug-resistant bacteria. | 312 (95.71) | 11 (3.37) | 3 (0.92) | 0 | 0 |
| A8. Due to your concern about the potential transmission of germs from the hospital to your family, you exercise great caution while at work. | 187 (57.36) | 101 (30.98) | 28 (8.59) | 4 (1.23) | 6 (1.84) |

**Note:** Item A1 was assigned a reverse score.

Supplementary **Table S3. Practice**

| Items | Very compliantly, n (%) | Relatively compliantly, n (%) | Neutral, n (%) | Relatively incompliantly, n (%) | Very incompliantly, n (%) |
| --- | --- | --- | --- | --- | --- |
| P1. Take care to separately place patients with multi-resistant bacterial infections from non-infected patients. | 274 (84.05) | 46 (14.11) | 4 (1.23) | 1 (0.31) | 1 (0.31) |
| P2. Wear a barrier gown when performing clinical procedures on patients with multidrug-resistant infections. | 267 (81.90) | 49 (15.03) | 9 (2.76) | 1 (0.31) | 0 |
| P3. Wear a face shield when you may be exposed to droplets or aerosols from patients with multidrug-resistant infections. | 216 (66.26) | 66 (20.25) | 27 (8.28) | 10 (3.070 | 7 (2.15) |
| P4. Wear gloves when coming into contact with wounds, blood, body fluids, drainage fluid, and secretions from patients with multidrug-resistant infections and will wash or disinfect your hands immediately after removing gloves. | 307 (94.17) | 13 (3.99) | 6 (1.84) | 0 | 0 |
| P5. When managing patients with multidrug-resistant infections in the clinical setting, you will strictly adhere to the principle of the exclusive use of specialized items. | 278 (85.28) | 43 (13.19) | 4 (1.23) | 0 | 1 (0.31) |
| P6. Pay close attention to the instruments and equipment used by patients with multidrug-resistant infections and have them disinfected at the end of the day by the relevant staff or by the patients themselves. | 292 (89.57) | 28 (8.59) | 4 (1.23) | 1 (0.31) | 1 (0.31) |
| P7. Treat a suspected person with multidrug-resistant infections as a multidrug-resistant patient until the results of the pathogenic microbiological tests are confirmed. | 254 (77.91) | 49 (15.03) | 22 (6.75) | 1 (0.31) | 0 |
| P8. You are fully aware of the prevention and control of multidrug-resistant infections and will strictly implement them in your clinical practice. | 202 (61.96) | 103 (31.60) | 20 (6.13) | 1 (0.31) | 0 |
| P9. Dispose of household waste and medical waste generated by patients with multidrug-resistant infections in double yellow medical waste bags. | 283 (86.81) | 32 (9.82) | 8 (2.45) | 1 (0.31) | 2 (0.61) |
| P10. Promptly advise and correct the inappropriate or wrong behavior by patients, their families, and other medical staff. | 225 (69.02) | 87 (26.69) | 13 (3.99) | 1 (0.31) | 0 |

Supplementary Table S4. Structural equation modeling.

| Pathways | β (95% CI) | P |
| --- | --- | --- |
| Knowledge → Attitude | 0.61 (0.48, 0.74) | < 0.001 |
| Knowledge → Practice | -0.30 (-0.52, 0.08) | 0.009 |
| Knowledge → Job Satisfaction | -0.02 (-0.05, 0.01) | 0.222 |
| Attitudes → Practice | 0.89 (0.73, 1.06) | < 0.001 |
| Job satisfaction → Attitude | 0.52 (0.05, 0.98) | 0.030 |
| Job satisfaction → Practice | 0.75 (0.03, 1.47) | 0.040 |
| Professional title → Job Satisfaction | 0.15 (0.05, 0.25) | 0.004 |
| Professional title → Knowledge | 0.35 (-0.01, 0.71) | 0.055 |
| Professional title → Attitude | -0.33 (-0.77, 0.11) | 0.137 |
| Professional title → Practice | -0.41 (-1.09, 0.26) | 0.230 |
| Clinical years → Knowledge | 0.17 (-0.04, 0.38) | 0.109 |
| Clinical years → Attitude | 0.11 (-0.15, 0.36) | 0.419 |
| Clinical years → Practice | 0.21 (-0.19, 0.60) | 0.306 |
| Clinical years → Job Satisfaction | -0.11 (-0.16, -0.05) | < 0.001 |
| Clinical years → Professional title | 0.35 (0.29, 0.40) | < 0.001 |
