# Supplementary material for: Knowledge, attitude, and practice towards bacterial multidrug-resistance and structural equation modeling analysis among intensive care unit nurses and physicians
Source: PLoS One. 2024 Jun 14;19(6):e0304734. doi: 10.1371/journal.pone.0304734 (PMC11178221; doi:10.1371/journal.pone.0304734)
Supplement: S1 Questionnaire — (DOCX) [file pone.0304734.s002.docx]

| Survey ID: | | | |
| --- | --- | --- | --- |
| Dear Healthcare Professional,  We are researchers from XX Hospital, inviting you sincerely to participate in our research project. The aim of this study is to understand the knowledge, attitudes, and practices of ICU healthcare personnel regarding multi-drug resistant bacteria. The findings will serve as a foundation for developing evidence-based intervention strategies, potentially benefiting a larger population and improving their health outcomes in the future. Your participation in this research is voluntary, and the study has been ethically reviewed and approved by the Ethics Review Committee. If you agree to participate, please refer to the following instructions.  1. Kindly complete the questionnaire; there are no right or wrong answers, and you only need to provide information based on your actual experiences. Feel free to reach out to us with any questions during the answering process, and please submit your responses promptly after completion.  2. This study involves a simple survey that will not cause harm to your physical or psychological well-being. However, it may touch upon some private information, such as your gender and age. We assure you that we will strictly maintain confidentiality and will not disclose your information. Please feel confident in providing this information.  3. As a participant, you can stay informed about the study's progress and related information. If you decide to withdraw from the study, please inform us, and your data will not be included in the research results.  Finally, we sincerely appreciate your valuable time and support for our scientific research!  □I am aware of and consent to the use of the collected data for scientific research.  Informed Consent Signature:  Participation Date: year month day | | | |
| **Part 1 basic information** | | | |
| **1.gender：** | | a. male | b. female |
| **2.age： years** | | | |
| **3.education：** | | a. high school/ technical school  b. college/ bachelor’s degree  c. master/PhD | |
| **4.nature of your workplace：** | | a. public primary/secondary  b. public tertiary  c. private Hospital | |
| **5.occupation：** | | a. physician  b. nurse | |
| **6.professional title：** | | a. junior and below  b. intermediate  c. vice senior/senior | |
| **7.years of clinical experience：** | | a. < 3  b. 3-4.9  c. 5-9.9  d. ≥ 10 | |
| **8.years of ICU work experience：** | | a. < 1  b. 1-2.9  c. 3-4.9  d. 5-9.9  e.≥10 | |
| **9.** **job satisfaction：** | | a. very satisfied  b. relatively satisfied  c. neutral  d. moderate satisfied  e. dissatisfactory | |

**Part 2 Understanding of Multi-Drug Resistant Bacteria**

| K1. The definition of multidrug-resistant bacteria. | | | | | | | | a. correct | | | b. wrong | | | c. unclear |  |
| --- | --- | --- | --- | --- | --- | --- | --- | --- | --- | --- | --- | --- | --- | --- | --- |
| K2. The main risk factors for multidrug-resistant bacterial infections: | | | | | | | |  | | |  | | |  |  |
| K2.1. Adolescents | | | | | | | | a. correct | | | b. wrong | | | c. unclear |  |
| K2.2. Immunocompromised (including patients with diabetes mellitus, chronic obstructive pulmonary disease, cirrhosis, uremia, and oncology patients on long-term immunosuppressive therapy, radiation, and/or chemotherapy) | | | | | | | | a. correct | | | b. wrong | | | c. unclear |  |
| K2.3. Undergoing invasive procedures, with prolonged tube placement | | | | | | | | a. correct | | | b. wrong | | | c. unclear |  |
| K2.4. Prolonged hospital stays | | | | | | | | a. correct | | | b. wrong | | | c. unclear |  |
| K2.5. With a history of multidrug-resistant bacterial colonization or infection | | | | | | | | a. correct | | | b. wrong | | | c. unclear |  |
| K2.6. Recently (within 90 days) treated with 3 or more antimicrobial drugs | | | | | | | | a. correct | | | b. wrong | | | c. unclear |  |
| K3. Patients and carriers of multidrug-resistant bacterial infections are the main sources of biological transmission of these bacteria in the hospital. | | | | | | | | a. correct | | | b. wrong | | | c. unclear |  |
| K4. Medical devices and the environment that is contaminated with multidrug-resistant bacteria constitute an abiotic source in hospitals. | | | | | | | | a. correct | | | b. wrong | | | c. unclear |  |
| K5. The transmission of multidrug-resistant bacteria in hospitals can be achieved in a variety of ways, while contact transmission being the most common method of transmission in hospitals. | | | | | | | | a. correct | | | b. wrong | | | c. unclear |  |
| K6. Wearing gloves when touching patients with multidrug-resistant infections can prevent hand contamination, and therefore hand washing is not required after removing gloves. | | | | | | | | a. correct | | | b. wrong | | | c. unclear |  |
| K7. Medical staffs should wear a face mask, mask, goggles, and isolation clothing when performing operations that may cause splashing of blood, body fluids, secretions, or excretions. | | | | | | | | a. correct | | | b. wrong | | | c. unclear |  |
| K8. Patients with multidrug-resistant infections or colonization should be placed in a single room as far as possible. If a single room is not available, can patients with the same multi-resistant infection or colonization be placed in the same room? | | | | | | | | a. correct | | | b. wrong | | | c. unclear |  |
| K9. Medical equipment, instruments, and items used by patients with multidrug-resistant infections, should be used exclusively by the patient and disinfected in a timely manner. | | | | | | | | a. correct | | | b. wrong | | | c. unclear |  |
| K10. Medical equipment or instruments used by patients with multi-drug resistant infections, which cannot be dedicated for exclusive use, should be thoroughly wiped and disinfected after each use. | | | | | | | | a. correct | | | b. wrong | | | c. unclear |  |
| K11. Wearing gloves when touching patients with multidrug-resistant infections can prevent hand contamination, and therefore hand washing is required after removing gloves. | | | | | | | | a. correct | | | b. wrong | | | c. unclear |  |
| **Part 3 Attitudes and Thoughts Regarding Multi-Drug Resistant Bacteria** | | | | | | | | | | | | | | | |
| A1. Multidrug-resistant infections are common in the ICU and are difficult to avoid, so there is no need to pay particular attention to them. | | a. strongly agree | | b. agree | | c. neutral | | | d. disagree | | | e. strongly disagree | | | |
| A2. Maintaining proper hand hygiene among medical staff is crucial for the prevention and control of multidrug-resistant bacteria infections. | | a. very important | | b. important | | c. neutral | | | d. less important | | | e. not important at all | | | |
| A3. Physicians must adhere to basic principles, implement hierarchical management, and use personalized medication of antibacterial drugs, which is important for prevention and control of multidrug-resistant bacteria infections. | | a. very important | | b. important | | c. neutral | | | d. less important | | | e. not important at all | | | |
| A4. Clinical nurses should possess a thorough understanding of multidrug-resistant bacteria and diligently implement measures to isolate, prevent and control their spread, which is important for prevention and control of multidrug-resistant bacteria infections. | | a. very important | | b. important | | c. neutral | | | d. less important | | | e. not important at all | | | |
| A5. Each medical staff should actively participate in the prevention and control of multidrug-resistant bacteria. | | a. strongly agree | | b. agree | | c. neutral | | | d. disagree | | | e. strongly disagree | | | |
| A6. It is necessary to strengthen the training and continuing education for clinical nurses regarding multidrug-resistant bacteria. | | a. strongly agree | | b. agree | | c. neutral | | | d. disagree | | | e. strongly disagree | | | |
| A7. It is necessary to strengthen the training and continuing education for physicians regarding multidrug-resistant bacteria. | | a. strongly agree | | b. agree | | c. neutral | | | d. disagree | | | e. strongly disagree | | | |
| A8. Due to your concern about the potential transmission of germs from the hospital to your family, you exercise great caution while at work. | | a. very concerned | | b. concerned | | c. neutral | | | d. less concerned | | | e. not concerned at all | | | |
| **Part 4 Behaviors and Practices Regarding Multi-Drug Resistant Bacteria** | | | | | | | | | | | | | | |  |
| P1. Take care to separately place patients with multi-resistant bacterial infections from non-infected patients. | a. very compliantly | | b. relatively compliantly | | c. neutral | | | d. relatively incompliantly | | | e. very incompliantly | | | |  |
| P2. Wear a barrier gown when performing clinical procedures on patients with multidrug-resistant infections. | a. very compliantly | | b. relatively compliantly | | c. neutral | | | d. relatively incompliantly | | | e. very incompliantly | | | |  |
| P3. Wear a face shield when you may be exposed to droplets or aerosols from patients with multidrug-resistant infections. | a. very compliantly | | b. relatively compliantly | | c. neutral | | | d. relatively incompliantly | | | e. very incompliantly | | | |  |
| P4. Wear gloves when coming into contact with wounds, blood, body fluids, drainage fluid, and secretions from patients with multidrug-resistant infections and will wash or disinfect your hands immediately after removing gloves. | a. very compliantly | | b. relatively compliantly | | c. neutral | | | d. relatively incompliantly | | | e. very incompliantly | | | |  |
| P5. When managing patients with multidrug-resistant infections in the clinical setting, you will strictly adhere to the principle of the exclusive use of specialized items. | a. very compliantly | | b. relatively compliantly | | c. neutral | | | d. relatively incompliantly | | | e. very incompliantly | | | |  |
| P6. Pay close attention to the instruments and equipment used by patients with multidrug-resistant infections and have them disinfected at the end of the day by the relevant staff or by the patients themselves. | a. very compliantly | | b. relatively compliantly | | c. neutral | | | d. relatively incompliantly | | | e. very incompliantly | | | |  |
| P7. Treat a suspected person with multidrug-resistant infections as a multidrug-resistant patient until the results of the pathogenic microbiological tests are confirmed. | a. very compliantly | | b. relatively compliantly | | c. neutral | | | d. relatively incompliantly | | | e. very incompliantly | | | |  |
| P8. You are fully aware of the prevention and control of multidrug-resistant infections and will strictly implement them in your clinical practice. | a. very compliantly | | b. relatively compliantly | | c. neutral | | | d. relatively incompliantly | | | e. very incompliantly | | | |  |
| P9. Dispose of household waste and medical waste generated by patients with multidrug-resistant infections in double yellow medical waste bags. | a. very compliantly | | b. relatively compliantly | | c. neutral | | | d. relatively incompliantly | | | e. very incompliantly | | | |  |
| P10. Promptly advise and correct the inappropriate or wrong behavior by patients, their families, and other medical staff. | a. very compliantly | | b. relatively compliantly | | c. neutral | | | d. relatively incompliantly | | | e. very incompliantly | | | |  |
